# Radiofrequency Thermal Ablation for a Small Papillary Thyroid Carcinoma in a Patient Unfit for Surgery: A Case Report

**DOI:** 10.3389/fendo.2021.566362

**Published:** 2021-03-29

**Authors:** Francesca Maletta, Sara Garberoglio, Alessandro Bisceglia, Alberto Ragni, Francesca Retta, Marco Gallo, Roberto Garberoglio, Mauro Papotti

**Affiliations:** ^1^ Pathology Unit, Department of Laboratory Medicine, Città della Salute e della Scienza Hospital, Turin, Italy; ^2^ Division of Endocrinology, Diabetology and Metabolism, Department of Medical Sciences, Città della Salute e della Scienza Hospital, University of Turin, Turin, Italy; ^3^ Oncological Endocrinology Unit, Department of Medical Sciences, Città della Salute e della Scienza Hospital, University of Turin, Turin, Italy; ^4^ Endocrinology and Metabolic Diseases Unit, AO S.S. Antonio e Biagio e Cesare Arrigo, Alessandria, Italy; ^5^ Pathology Unit, Department of Oncology, University of Turin, Turin, Italy

**Keywords:** radiofrequency, ablation, papillary carcinoma, thyroid, fine needle aspiration, minimally invasive procedure, case report

## Abstract

Ultrasound-guided radiofrequency thermal ablation has been proposed as an effective and safe procedure for treating patients who have low-risk papillary thyroid microcarcinomas and/or are unfit for surgery. We present the case of a 72-year old male patient with a small thyroid nodule diagnosed as papillary carcinoma after fine needle aspiration. Since the patient had other serious comorbidities, priority was given to other therapies and the malignant thyroid nodule was submitted to active surveillance. After detecting at a follow-up examination a slight dimensional increase of the nodule, the possibility of a radiofrequency thermal ablation was proposed to our patient, who accepted. The procedure was safely and effectively carried out. Follow-up examinations with ultrasonography (or contrast enhanced ultrasound), conducted after 1, 3, 6, and 12 months, demonstrated a progressive reduction of size and loss of vascularization in the treated area. The fine needle aspiration was repeated after 6 months: the sample revealed a very poor cellularity composed of inflammatory cells and thick colloid; no residual neoplastic cells were observed. Our experience confirmed what already demonstrated by previous reports: radiofrequency ablation can effectively eliminate small papillary carcinomas, with a very low complication rate. It may be an alternative strategy for the treatment of low-risk, indolent papillary thyroid microcarcinomas, thus avoiding the potential side-effects of surgery in patients at risk for relevant comorbidities.

## Introduction

Surgery represents the first approach for the treatment of papillary thyroid carcinoma (PTC). However, considering the overall good prognosis of this neoplasm, extensive radical resection may not be indicated for some patients due to the risk of surgical complications and impaired quality of life in the postoperative period (1, 2). For years, radiofrequency ablation (RFA) has been considered a safe and effective method for the treatment of benign thyroid nodules and recurrent thyroid tumors. Currently, several guidelines recommend RFA treatment for symptomatic benign thyroid nodules, while indications for the treatment of malignant nodules are limited to palliative treatment in recurrent thyroid tumors or metastatic lymph nodes, when surgery is contraindicated or refused by the patient (1–5). RFA has also been recently suggested as an alternative treatment modality for primary thyroid microcarcinomas (1–3, 6) and in 2017, the RFA guidelines of the Korean society of thyroid radiology proposed the use of RFA for patients with primary thyroid cancer who refuse or are unfit for surgery (7).

## Case Description

**Figure 1:**
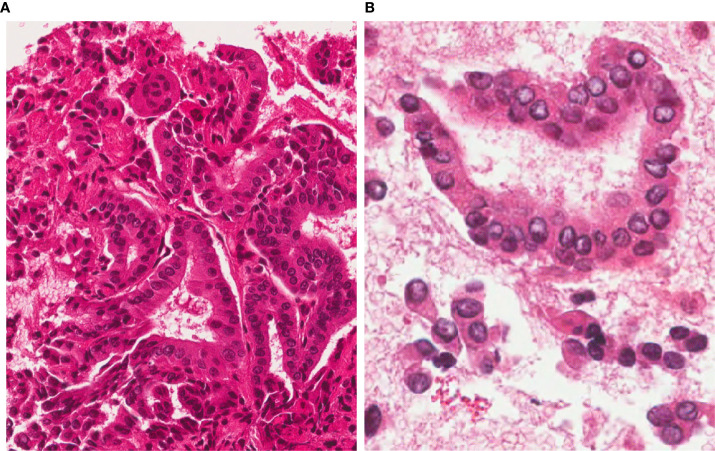
FNAC before RFA. 4 µm-thick H&E stained sections from the cell-block show a highly cellular sample, with a typical papillary architecture; thyrocytes show irregular, large and clear nuclei. A diagnosis of Papillary Thyroid Carcinoma (TIR5 according to the Italian SIAPEC-IAP classification of thyroid cytology) was formulated (**A**, magnification x200; **B**, x400).

A 72-year old male patient was referred to our clinic after the diagnosis of a left axillary lymph node metastasis from Merkel cell carcinoma (MCC) with high mitotic activity and proliferation index. A dermatologic evaluation showed no sign of a primary skin lesion. A ^18^F-fluoro-2-deoxy-d-*glucose Positron Emission Tomography (* ^18^FDG-PET)/Computed Tomography (CT) scan was performed for initial tumor staging and revealed an uptake in the left axilla (site of the known lymph node lesion) and a focal uptake in the left thyroid lobe. The subsequent thyroid ultrasound (US) showed, in the medial third of the left thyroid lobe, an 8x8x7 mm markedly hypoechoic, solid, subcapsular nodule with irregular margins and microcalcifications (high-risk category according to the 2016 AACE/ACE/AME US classification system) (8). No suspicious cervical lymph-nodes were detected. TSH and calcitonin levels were normal. Given the suspicious US features and the ^18^FDG-PET uptake, a US-guided fine needle aspiration cytology (FNAC) was performed, being consistent with PTC (Tir 5 according to SIAPEC/IAP classification of thyroid cytology or category VI according to the Bethesda System) (9, 10) (**Figure 1**).

In consideration of the clinical aggressiveness of MCC and its staging, priority was given to the treatment of MCC and a strategy of active surveillance for the small malignant thyroid nodule was chosen.

Subsequently, patient underwent external beam radiotherapy treatment to the axillary lesion (50 Gy/25 fractions), and, after detection of metastatic liver lesions and peri-pancreatic lymph node metastases, multiple cycles of systemic Carboplatin/Etoposide chemotherapy were administered. After a further disease progression, the patient was switched to an immunotherapy-based, second-line systemic treatment regimen with avelumab. The following restaging CT scans showed progressive reduction in the size of known liver metastases, confirming a partial response to the immunotherapy regimen, which was then discontinued after 18 months due to stable disease.

During the US follow-up exams, the malignant thyroid nodule showed a slight dimensional increase, reaching the size of 11x9x8 mm of maximum diameter. Taking into consideration patient’s age, performance status, the overall clinical situation and surgical risk, a less invasive treatment approach was preferred, and after obtaining patient’s informed consent, a procedure of RFA of the malignant thyroid nodule was performed.

## Description of the Procedure

The treatment of RFA was carried out in day hospital regimen. A peripheral vein was cannulated in the forearm with a venous catheter. During the procedure, 500 ml saline and 50 mg of Ranitidine were administered. The patient was connected to a monitor which allowed to check peripheral oxygen saturation, electrocardiogram, respiratory rate and blood pressure during the whole duration of the procedure. He was placed supine with his neck extended, in order to allow a better exposure of the jugular region.

**Figure 2:**
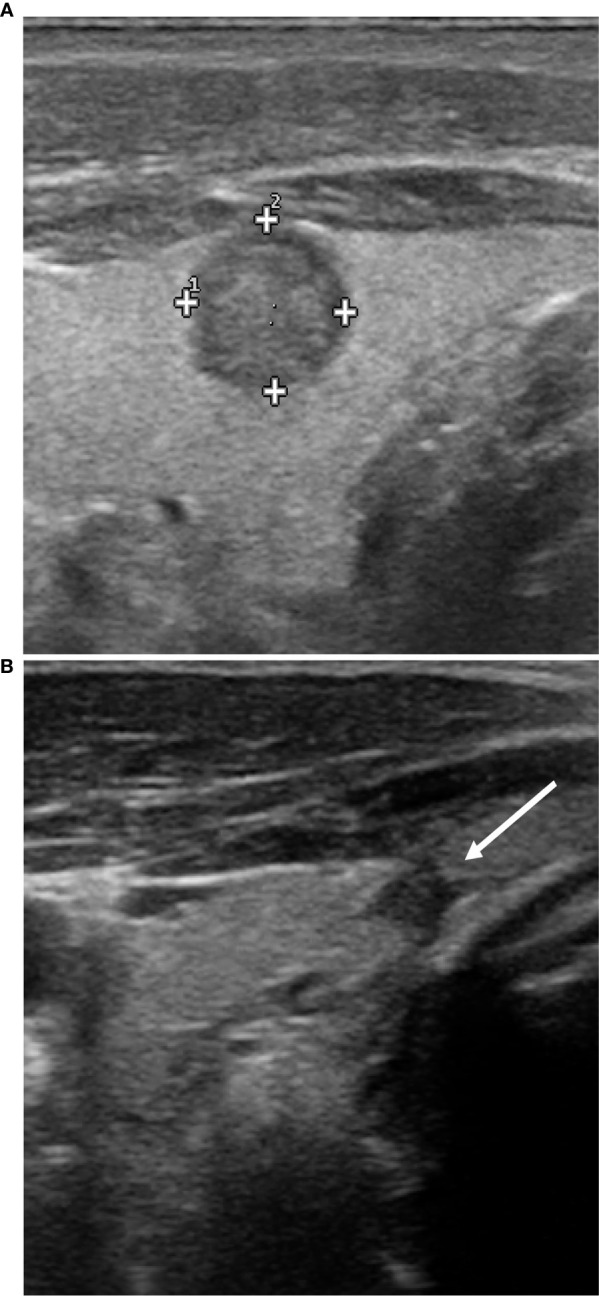
Ultrasound features of the nodule before and after RFA. Before RFA **(A)**, the nodulation has a solid, irregular, hypoechoic structure, with slightly spiculated margins and microcalcifications. At control 12 months after RFA only a small nodular, hypoechoic and homogeneous area is appreciable **(B)**; it is in contact with the thyroid capsule that appears slightly retracted but with no signs of extra-thyroidal extension.

The nodule was assessed by ultrasound: it was sub-capsular and localized in the left thyroid lobe, in the para-isthmic region; its diameters were 11x9x8mm. The echostructure was solid, irregular, hypoechoic, with slightly spiculated margins and with microcalcifications (**Figure 2**). At the Echo Color Doppler (ECD) investigation, vascularization was mainly endonodal; on strain elastosonography it appeared mainly rigid. The investigation was completed with the contrast-enhanced US (CEUS) (SonoVue Bracco, Milan) which allowed the complete visualization of the nodule vascular micro-network: it appeared mainly endonodal but irregular, with a scarcely vascularized central nucleus.

The procedure was carried out by RG, a collaborator of our group with at least 30 years of diagnostic and interventional ultrasound experience, especially of the neck region. After disinfecting the skin and setting up the sterile field, the suitable point was identified by US to proceed with local anesthesia with 2% lidocaine: the anesthetic was injected at the site of the skin puncture and in the space between the thyroid capsule and the fascia of the peri-thyroid muscles (strap muscles), where the algogenic endings of the sensory nerves are located.

The needle for RFA (needle used RFT-0710N RF, Medical Co.Ltd) was then inserted, under US guidance, with a trans-isthmic approach, always displaying the needle along its major axis. This type of approach allows to constantly monitor the needle in the context of the nodule, keeping it away from the recurrent laryngeal nerve, in order to reduce the risk of thermal injury (1–3). For the RFA procedure, a maximum power of 50 Watts was used.

As a rule, the technique of ablation consists in ideally dividing the nodule into smaller units that are independently treated using the “moving shot” technique: the tip of the electrode is initially positioned in the deeper portion with subsequent retractions towards the more superficial portions.

Given the small size of the nodule, the procedure was carried out quickly, with a duration of 5’11’’.

At the end of the procedure, before removing the needle, another CEUS examination was performed to assess the extension of the ablated area and compare it to the pre-treatment situation: the ablation proved complete and the ablated area was clearly wider than the edge of the previously assessed neoplasm.

After the procedure, the observation period lasted about 6 hours, with infusion of Methylprednisolone (125 mg), Acetaminophen (1 g) and Ranitidine (50 mg) in 500 ml saline solution. A last US exam of the anterior cervical region was performed to assess the absence of complications and then the patient was discharged home.

## Follow-Up

The patient was reassessed with the US at 1, 3, 6, and 12 months after the procedure, making a multiparametric US evaluation and adding the CEUS in the 3^rd^ and 6^th^ month controls.

At the examination performed after 1 month, the nodule had an oblong shape, measured approximately 13 x 22 x 9 mm (respectively, transverse, lateral and antero-posterior diameters) (T x L x AP) and had a solid, hypoechoic, slightly inhomogeneous echostructure, with regular margins. In the control performed after 3 months, the nodule had a slightly oblong shape, with a substantially unchanged volume from the previous exam, with measurements of approximately 12 x 18 x 8 mm (T x L x AP). The nodule had a solid, slightly irregular and hypoechoic structure. CEUS assessed, the described area appeared clearly avascular.

In the control performed after 6 months, the nodule had clearly reduced dimensions, always oblong in shape with dimensions of approximately 8 x 12 x 6 mm (T x L x AP), with a hypoechoic, uneven echostructure, with a small anterior marginal hyperechoic area.

When investigated by CEUS, the nodular area appeared to be totally avascular with less clear vascular margins compared to the previous examination. During this evaluation, FNAC with thin needle (22 Gauge) was performed. The sample was partly smeared on two slides, of which one was immediately alcohol-fixed and stained with Hamatoxylin and Eosin (H&E) for rapid on site evaluation while the other was air-dried and stained with Giemsa afterwards; the remaining sample in the syringe was collected and alcohol-fixed in a test tube. From this material, a paraffin-embedded cell-block was obtained, from which 5-µm thick sections were cut and stained with H&E.

**Figure 3:**
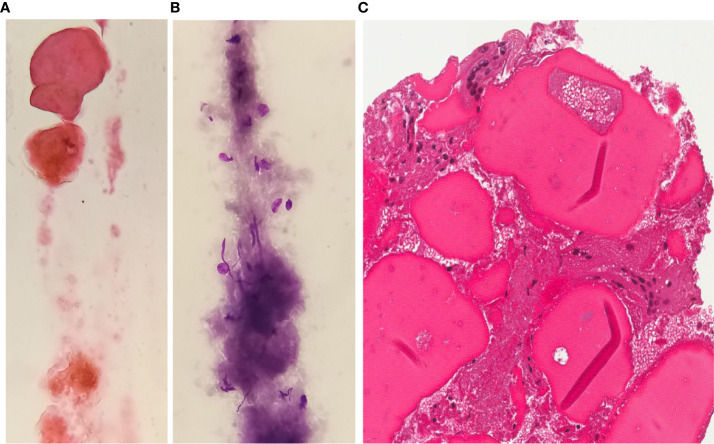
FNAC after RFA H&E **(A)** or Giemsa **(B)** stained smears and H&E stained section from cell-block **(C)** exhibit a poorly cellular sample with sparse inflammatory elements or multinucleated giant cells in a background of thick colloid and fibrosis (magnification x400).

The FNAC (both smears and cell-block) yielded a poorly cellular sample containing inflammatory cells (histiocytes and multinucleated cells), necrotic debris, fibrotic tissue, thick colloid and scattered thyrocytes, the latter showing no significant nuclear atypia (regular nuclear margins without groovings or pseudoinclusions) (**Figure 3**). If compared with the pre-RFA procedure FNAC, cellularity was considerably reduced and the typical signs of papillary carcinoma were totally absent.

After 12 months, only a small nodular area in the site of the previous treatment was appreciable, with a maximum size of about 4 mm, hypoechoic, homogeneous, without significant vascularization under ECD investigation (**Figure 2**). The nodule was in contact with the thyroid capsule that appeared slightly retracted, but seemed confined within the thyroid parenchyma, with no sign of extra-thyroidal extension. No pathological cervical lymph-nodes were found.

Nowadays, two years after the diagnoses of MCC and thyroid cancer, the patient is alive and well, substantially free of disease.

## Discussion

Since the first reported series in 2006 (11, 12), a number of studies showed the efficacy and safety in treating benign ‘cold’ and ‘hot’ thyroid nodules with RFA. Currently, several guidelines recommend RFA for generally benign thyroid nodules for symptomatic or cosmetic reasons and until recent years, recommendations for malignant diseases were restricted to palliative treatment for recurrent thyroid cancers or metastatic lymph nodes when surgery was contraindicated or declined by the patient (13–15).

However, several previous studies also showed that image-guided RFA is safe and effective in the treatment of primary thyroid microcarcinoma (PTMC) (16, 17) and this procedure was included in the 2017 RFA Guidelines of the Korean Society of Thyroid Radiology as an alternative to surgery for patients who refuse it or who cannot undergo an operation (7). Satisfactory results of image-guided thermal ablation for primary thyroid cancer were also reported using means other than radiofrequency, like laser and microwave (18, 19) but whether there is a difference among the efficacy of different techniques remains unclear and should be investigated with further studies.

As previously reported, after about two years of follow up, no sign of PTC recurrence was seen in our patient. This, together with the anatomo-pathological findings 6 months after the procedure, with no evidence of typical PTC cells, testifies the effectiveness of RFA in the treatment of malignant thyroid nodules. However, it should be considered that the patient was concomitantly taking avelumab; even if nowadays there are no data about the use of this drug in PTC treatment, there is a growing evidence regarding the efficacy of immunotherapy, and in particular of pembrolizumab, in the field of advanced thyroid cancer treatment (20). Therefore, we cannot exclude that this factor may also have contributed to the successful control of PTC in this patient)

The present case report recaps on some topics about RFA. As described elsewhere (1), the procedure starts by positioning the electrode into the deepest conceptual ablation unit of the nodule and under continuous US guidance and using the “moving shot” technique, the output RF power is administered until all conceptual units of the nodule have been covered. Our experience highlights an important concept about the technique of RFA: when treating a malignant nodule, thermoablation-induced necrosis must be extended beyond the borders of the nodule, even if it is in contact with the capsule, in order to be sure of the total ablation of the nodule. The US (morphological and contrast-enhanced) checks carried out in our case showed a tissue necrosis wider than the area of the tumor, thus testifying a positive result of the procedure. The tumor area was totally ablated and, at the end of the treatment, it appeared very small and avascular. However, attention must be paid not to damage the surrounding noble structures and in some cases it may be useful to interpose some liquid between the muscle band and the capsule. In the event that the nodules are in a position very close to the thyroid capsule or to the trachea, esophagus or arterial vessels, it is possible to perform a local infusion of liquid (saline solution, or 5% glucose), in order to create a liquid barrier between the nodule to be treated and the surrounding anatomical structures to protect the noble structures of the neck (the so-called “hydrodissection technique”). In our case, due to the position of the target nodule, it was not necessary to recur to this technique.

Contrary to the growing experience in using non-surgical procedures for thyroid nodule treatment (ethanol ablation, laser ablation, RFA, high-intensity focused ultrasound), morphological changes produced by ablation are rarely investigated apart from occasional descriptions of minimally-invasive techniques such as core needle biopsies (CNB) on residual nodule detected during follow-up after these procedures. In 2016, Branovan et al. (21) analyzed the gross and microscopic alterations in human thyroid tissue induced by RFA: the experiment was conducted on 37 thyroid glands surgically removed for follicular adenoma or adenomatous colloid goiter. After dividing the nodules into two parts, one was a subject for histological routine processing, the other one was used for the RFA procedure. On the half nodule treated with RFA, pathological examination revealed destructive and diffuse alterations in shape and size of parenchymal structures (follicles), stroma and vessels. Moreover, extensive necrotic areas, wrinkling of the tissue, swelling and blurring of cell details were observed. Also, larger vessels (venules) appeared spastic, while smaller ones (small lymphatics) were dilated.

Valcavi et al. (19) investigated the pathological effects of US-guided thermal laser ablation in three papillary microcarcinomas. The patients underwent percutaneous laser ablation and, subsequently, total thyroidectomy: conventional histology showed destructured and carbonized tissue, with no viable cells in the ablated area and in the rim of normal tissue surrounding the tumor. Similar results were obtained by Piana et al. (22) who evaluated the histopathological effects on 22 benign nodules treated with laser ablation and compared the cytological findings before and after the treatment with the histological features on surgical specimens.

Moreover, Zhang et al. (23) reported a case series of 98 patients treated with RFA on low-risk PTC. The follow-up CNB was performed in all patients after 3 months from the procedure, both in the ablation area and in the surrounding parenchyma and it showed degeneration of follicular epithelial cell, fibrosis with hyaline degeneration of interstitial collagen fibers and inflammatory lymphocytic infiltration at the center and at the edge of the ablation area. No residual or recurrent tumors were detected, which confirmed complete elimination of the PTMC lesions. These data were confirmed by a larger cohort of the same group (1), with CNBs showing degenerative changes such as edema, inflammation and sclerosis. Recently, Xiao et al. (24) evaluated the efficacy and safety of US-guided RFA for treating low-risk T1bN0M0 PTC. US-guided CNB was routinely performed at the center and edge of the ablation zone as well as in the surrounding thyroid parenchyma 3 or 6 months after ablation; among the ablation zones that did not disappear (28 cases on a total of 66 patients), CNB revealed no viable neoplastic cells in 26 cases, while two patients were found to have malignant cells on CNB at the edge of their ablation zones after 6 months of follow-up; these patients successfully underwent a second RFA session. A metastatic lymph-node was detected at ipsilateral level IV in one patient 3 months after ablation and this lymph-node was successfully treated with RFA. No distant metastases were detected during follow-up.

To our knowledge, our case is the first report on a FNAC (instead of a core biopsy) on a PTC nodule treated with RFA: results are similar to those shown in CNBs by Zhang et al. (23) and Wu et al. (1), namely a sample showing prevalent sclerosis, inflammation and absence of residual neoplastic cells. Thus, traditional FNAC may prove as effective as CNB in order to assess complete response to RFA or to detect a possible residual disease. Moreover, it has the advantage of being a less invasive procedure than CNB: CNB needles in fact are larger in caliber than FNAC needles and they should be used only by experienced operators; less-experienced operators may have difficulties in tracking the needle tip under US, thus increasing the possibility of complications (25).

In conclusion, image-guided thermal ablation of low risk PTC is becoming a widely accepted procedure: with the advantage of minimal invasiveness, it might be chosen as first line treatment for patients unfit for surgery or to avoid its complications. Some authors even suggest that image-guided thermal ablation might be a good option to compensate for image-derived cancer overdiagnosis (26), with PTCs being more and more often detected in their very initial stage due to improved imaging techniques.

However, so far, some limitations to the widespread use of RFA for treating PTMC still exist: first of all, available data are based on retrospective studies only, with inevitable sample bias (1). Moreover, considering the good prognosis of PTC, definitive conclusions about the efficacy of RFA might be drawn only after longer term follow-up, not yet available at the present moment.

## Data Availability Statement

The original contributions presented in the study are included in the article/supplementary material. Further inquiries can be directed to the corresponding author.

## Ethics Statement

Written informed consent was obtained from the individual for the publication of any potentially identifiable images or data included in this article.

## Author Contributions

FM and SG conceived the idea of this essay. SG, AB, AR, FR, MG, and RG participated in the treatment and collected the case history. FM made the cytological diagnoses. FM, SG, and RG wrote the case report. RG and MP revised the manuscript.

## Funding

Work supported by grants from AIRC, Milan (IG n. 20100 to MP).

## Conflict of Interest

The authors declare that the research was conducted in the absence of any commercial or financial relationships that could be construed as a potential conflict of interest.
